# Prenatal iron supplementation adjusted to maternal iron stores reduces behavioural problems in 4‐year‐old children

**DOI:** 10.1111/mcn.13595

**Published:** 2023-12-02

**Authors:** Lucía Iglesias‐Vázquez, Josefa Canals, Carmen Hernández‐Martínez, Núria Voltas, Victoria Arija

**Affiliations:** ^1^ Department of Basic Medical Sciences, Nutrition and Mental Health (NUTRISAM) Research Group Universitat Rovira I Virgili Reus Spain; ^2^ Institut d'Investigació Sanitaria Pere Virgili (IISPV) Reus Spain; ^3^ Department of Psychology, Research Centre for Behavioral Assessment (CRAMC), Faculty of Education Sciences and Psychology Universitat Rovira I Virgili Tarragona Spain; ^4^ Department of Psychology, Faculty of Education Sciences and Psychology, Serra Húnter Fellow Universitat Rovira I Virgili Tarragona Spain; ^5^ Collaborative Research Group on Lifestyles, Nutrition, and Smoking (CENIT), Tarragona‐Reus Research Support Unit IDIAP Jordi Gol Tarragona Spain

**Keywords:** behavioural problems, child behaviour, executive functioning, iron supplementation, pregnancy

## Abstract

Prenatal iron supplementation improves children's health and cognitive performance, but few studies explore behavioural development. This study assessed the effects of adjusting prenatal iron supplementation to maternal iron stores during early pregnancy on children's behavioural problems. Randomized controlled trial conducted in Tarragona (Spain) involving 230 nonanaemic pregnant women and their children after a 4‐year follow‐up. Based on haemoglobin (Hb) levels before gestational week (GW) 12, women receive different iron doses: those with Hb = 110–130 g/L were randomized to receive 80 or 40 mg/day and those with Hb > 130 g/L were randomized to receive 20 or 40 mg/day. Maternal iron stores at GW12 were classified using serum ferritin (SF) as low (SF < 15 µg/L), normal (SF = 15–65 µg/L), and normal‐high (SF > 65 µg/L). Children's behaviour was assessed by parents using the Child Behaviour Checklist for ages 1.5–5 years and the Behaviour Rating Inventory of Executive Function‐Preschool Version, and by teachers using the Teacher's Report Form for ages 1.5–5 years. Multivariable regression models were performed. Taking 80 mg/day of iron improved child behaviour when women had low iron stores but worsened it when mothers had normal–high iron stores, except for depressive and attention/hyperactivity problems. Taking 20 mg/day of iron improved behaviour only in those children whose mothers had SF > 65 µg/L in early pregnancy. Additionally, executive functioning improved at high doses of prenatal iron when maternal baseline SF < 15 µg/L. Adjusting prenatal iron supplementation to both maternal baseline Hb levels and iron stores reduces behavioural problems in 4‐year‐old children.

## Key messages

- Prenatal iron supplementation succeeds in improving maternal haematological outcomes and cognitive performance of children, but few studies have focused on behavioural development.
- Prenatal iron supplementation adjusted to maternal iron stores reduces behavioural problems in 4‐year‐old children.
- Maternal iron stores early in pregnancy should be measured in addition to haemoglobin concentration.
- These findings contribute greatly to the advancement of public health by giving physicians the tools to prescribe the most appropriate dose of iron supplementation to contribute to better child development.

## Graphical abstract

In pregnant women without anaemia, high‐dose prenatal iron was effective in reducing the risk of behavioural problems in children of mothers with early pregnancy iron deficiency. Conversely, low‐dose prenatal iron was effective in reducing the risk of behavioural problems in children of mothers with high iron reserves.

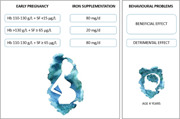

## 1 INTRODUCTION

Society is currently facing an epidemic of mental health problems, with a worrying increase in the prevalence of behavioural and psychological problems in childhood (Barican et al., 2022). Despite the multifactorial aetiology of psychopathological disorders, the intrauterine environment is one of the factors with which they are strongly associated (DiPietro et al., 2018; Feldman, 2008; Takegata et al., 2021; Tearne et al., 2015). On the one hand, behaviour has its physiological basis in the brain's structure, whose formation and maturation begin during the foetal phase (Cusick & Georgieff, 2016). Proper brain development requires iron as an essential element for many physiological events, so maternal iron status during pregnancy plays an important role in this process (Georgieff, 2007; McCann et al., 2020; Quezada‐Pinedo et al., 2021).

Prenatal iron deficiency (ID) has been associated with poorer cognitive development, intelligence quotient (IQ), and executive functions in children, even in mild stages without signs of anaemia (Gaillard et al., 2014; Hernández‐Martínez et al., 2011; McCarthy et al., 2021; Radlowski & Johnson, 2013). Initial evidence of an association between maternal iron status and children's behaviour has also been recently provided (Díaz‐López et al., 2022; Hernández‐Martínez et al., 2011; Iglesias et al., 2017). In a large Spanish cohort of mother–child pairs, high ferritin levels during pregnancy showed a protective effect against inattentive‐type attention‐deficit/hyperactivity disorder (ADHD) symptoms assessed at 4 years of age (Santa‐Marina et al., 2020). Further, some studies have referred to specific periods during pregnancy of greater vulnerability to ID regarding child outcomes, although the evidence is inconsistent. On the one hand, a recent systematic review concluded that low iron, particularly in the third trimester of gestation, may be associated with adverse offspring neurodevelopment (Janbek et al., 2019). On the other hand, population registers data from the Stockholm Youth Cohort indicated that iron deficit early in pregnancy was associated with an increased risk of a diagnosis of autism spectrum disorders and ADHD in offspring (Wiegersma et al., 2019). Furthermore, neonatal ID assessed by umbilical cord serum ferritin (SF) concentrations, which correlate with maternal iron status, has been associated with lasting behavioural consequences at 5 years (McCarthy et al., 2021).

According to previous findings, prenatal iron supplementation is effective in improving maternal haematological outcomes (Peña‐Rosas et al., 2015; Vázquez et al., 2019), but very little effort has been devoted to assessing its effect on child neurodevelopment. In this regard, previous data from the ECLIPSES study have shown that adapting prenatal iron supplementation to maternal needs based on haemoglobin (Hb) and SF concentrations improves the child's cognitive functioning at 4 years of age (Iglesias‐Vázquez et al., 2023). Other areas of neurodevelopment, such as behavioural problems are still poorly investigated. As for the development of behavioural problems in infancy, only one study appears to have addressed this question, without being able to provide clear evidence of the benefit of prenatal iron supplementation on the behaviour of children aged 4 years (Zhou et al., 2006) and 6–8 years (Parsons et al., 2008). In contrast, the authors found a slightly higher percentage of children with abnormal behaviour at both ages among those whose mothers received iron compared with those who received a placebo (Szajewska et al., 2010). Although cognitive functioning and behavioural performance are part of an overall neurodevelopmental process, they are different aspects of it. Thus, as delays in cognitive and behavioural functions may or may not co‐occur, with varying consequences for children, it is worth assessing each aspect individually. As it is known that many biological and lifestyle conditions could influence maternal iron status (Hanson, 2001; Quezada‐Pinedo et al., 2022; Rasmussen et al., 2005), the present study aimed to assess the effect of prenatal iron supplementation in non‐anaemic pregnant women by adjusting the dose to the mother's actual iron needs on their children's risk of behavioural problems at 4 years of age.

## 2 METHODS

### 2.1 Study sample

The ECLIPSES study was a population‐based randomized controlled trial conducted in 2013–2017 in Tarragona, Spain, which aimed to evaluate the effectiveness of different doses of prenatal iron supplementation on maternal iron status at the end of pregnancy in nonanaemic women in early pregnancy (Vázquez et al., 2019). Secondary objectives also included the assessment of the effectiveness of different doses of prenatal iron supplementation on the cognitive (Iglesias‐Vázquez et al., 2023) and behavioural development of their children. The current analyses aimed to assess the effect of that supplementation on child behavioural development and their risk of having behavioural problems at 4 years of age.

**Figure 1:**
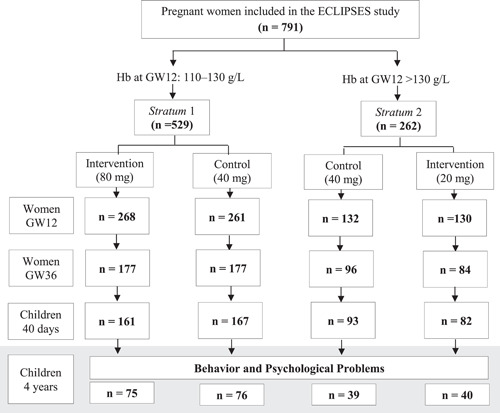
Flowchart of the study. GW, gestational week; Hb, haemoglobin.

Participating women were recruited before the gestational week (GW) 12 and allocated into two groups according to their Hb levels at the time. Women in Stratum 1 (initial Hb = 110–130 g/L) were randomly prescribed a daily dose of 40 or 80 mg of iron aiming to prevent an iron deficit, whereas those in Stratum 2 (initial Hb > 130 g/L) received 40 or 20 mg of iron daily aiming to prevent the risk of developing iron excess (Figure 1). As the usually prescribed dose of iron is daily 40 mg, women in each Strata receiving that dose were considered the control group against which the higher and lower iron dose interventions were tested. The intervention was triple‐blind, meaning that neither the researchers, the supplement providers, nor the health workers knew each woman's dose of iron supplements until the end of the study. Compliance with the intervention was done by comparing the number of leftover pills that participants return in each visit with the self‐reported compliance and was considered ‘good’ when women forgot to take the pill less than twice a week, and ‘low’ when they forgot it two or more times a week at any of the study visits. The ECLIPSES study was registered at www.clinicaltrialsregister.eu as EudraCT number 2012‐005480‐28 and its methodological details can be found extended elsewhere (Arija et al., 2014; Vázquez et al., 2019).

### 2.2 Measures

Behavioural and emotional problems of children at age 4 years were reported by parents using the Child Behaviour Checklist for ages 1.5–5 years (CBCL1½‐5) (Achenbach & Rescorla, 2000) and by teachers using the Teacher's Report Form for ages 1.5–5 years (TRF1½‐5) (Achenbach & Rescorla, 2000). The CBCL1½‐5 and TRF1½‐5 are tests of 99 items (with three response options: not true; somewhat or sometimes true; very true or often true), which provide six empirically based syndrome scales (emotional reactivity, anxiety/depression, somatic complaints, withdrawal, attention problems and aggressive behaviour) and DSM 5‐oriented scales (depressive problems, anxiety problems, autism spectrum problems, attention‐deficit/hyperactivity problems and oppositional defiant problems). Emotional reactivity, anxiety/depression, somatic complaints and withdrawal scales constitute the scale of the internalizing problems, whereas the attention problems and aggressive behaviour scales constitute the scale of the externalizing problems. All the syndromic scales together constitute the total problem scale. T‐scores for the Spanish version for all scales were used. Scores <65 are within the normal range, scores between 65 and 69 are borderline and scores >69 are in the clinical range. Internal consistency of the Spanish version covered the range of moderate to good (de la Osa et al., 2016).

The Behaviour Rating Inventory of Executive Function‐Preschool Version (BRIEF‐P) (Gioia et al., 2016) is a test for the evaluation of daily behavioural and observable aspects of the executive functions of children between 2 and 5 years old. This test of 63 items was answered by the parents, who must indicate with a Likert scale (never, sometimes, frequently) the frequency of certain problematic behaviours. The BRIEF‐P finally accounted for the following executive functions: inhibition, flexibility, emotional control, working memory and plan/organize; and allow to obtain four indexes: behavioural regulation index, flexibility index, metacognition index and global executive index. T‐scores (mean 50, SD 10) were used, with higher scores being equivalent to higher levels of executive dysfunction. The Spanish‐adapted version of the BRIEF‐P was used, which showed good data of reliability (Cronbach's *α* between 0.77 and 0.95 for the several scales) and validity (Gioia et al., 2016).

Women were visited once in each trimester of pregnancy and a wide range of information was recorded. Clinical and obstetric history was requested, including maternal age, parity and pregnancy planning. Anthropometric measurements (weight and height) were taken and body mass index was calculated. Dietary habits were assessed using a self‐administered food frequency questionnaire previously validated in our population (Rodríguez et al., 2008). In this process, participants retrospectively provided information on their usual food consumption at Weeks 12, 24 and 36 of pregnancy, 40 days after delivery and at follow‐up when the children were 4 years old. The food frequency questionnaire was reviewed and analysed by trained dietitians, who calculated daily food intake, energy content and various nutrients (Aparicio et al., 2020). Maternal lifestyle information included smoking and physical activity. Family socioeconomic status was calculated using the participants’ and partners’ educational level and occupational status (Vázquez et al., 2019). The parental IQ approach was assessed using the Matrix Reasoning subscale of the Wechsler Adult Intelligence Scale‐4th edition (Wechsler, 1997). Maternal anxiety status, measured by the State‐Trait Anxiety Inventory, and postpartum depression, assessed by the Edinburgh Postnatal Depression Scale, provided information about women's emotional status during pregnancy and after delivery. The State‐Trait Anxiety Inventory test assessed two separate concepts of anxiety: ‘state’ and ‘trait’. We included state anxiety as a covariate, which assesses a transient emotional state characterized by subjective, consciously perceived feelings of alertness and apprehension and autonomic nervous system hyperactivity. Detailed information can be found elsewhere (Vázquez et al., 2019).

In each trimester of pregnancy, blood samples were collected for biochemical determinations of Hb, SF and C‐reactive protein. Plasma polyunsaturated fatty acids (PUFAs) and serum vitamin D concentrations were quantified, because they are involved in brain development, so maternal levels during pregnancy may affect fetal neurodevelopment (Voltas et al., 2020; Zou et al., 2021). Folate and vitamin B_12_ measurements were also performed in the first trimester of pregnancy. Red blood cell (RBC) folate concentrations were then calculated using the following formula: (serum folate in haemolysed whole blood × dilution factor in haemolysis × 100)/haematocrit. Genetic determinations of *HFE* gene mutations were performed.

As for information about the children, the following data were recorded. At birth, sex, gestational age (calculated from the time since the first day of the last menstruation) and Apgar test score were obtained. Anthropometric measurements were recorded at birth and 40 days of age (length, weight and head circumference) and repeated at 4 years of age (weight and height). Similarly, the feeding mode was recorded at birth and 40 days of age and children's diet at 4 years of age was reported by parents using the same food frequency questionnaire as for participating women. Children's IQ was obtained at 4 years of age. All children were schooled by the time of the assessment.

### 2.3 Statistical analyses

Analyses were performed per protocol and stratified first according to baseline Hb concentrations following the study design, and then within each Stratum according to women's baseline iron stores, defined by their SF levels (SF < 15 µg/L, ID; SF = 15–65 µg/L, adequate iron stores; SF > 65 µg/L, normal‐high iron stores). The SF threshold for normal‐high iron stores corresponded to the 85th percentile.

Bivariate analyses of the variables studied were performed using the Student's *T* and the analysis of variance tests for continuous variables and the *χ* ^2^ test for categorical variables. The natural logarithm (Ln) transformation was applied to the SF concentration to normalize its distribution. For statistically significant results, the effect size was assessed using Cohen's *D*. Due to the multiple comparisons, the Bonferroni correction was applied to control the increase in Type I error. Multivariable regression models provided estimates of the effect of different doses of prenatal iron supplementation (Stratum 1: 80 mg vs. control, Stratum 2: 20 mg vs. control) on the child's behavioural problems. The models were adjusted a priori for those covariates that might influence this association. Maternal covariates include family socioeconomic status (low, middle, high), smoking (yes or no), parental IQ approach, energy intake, dietary intake of iron and nutrients related to its metabolism (fibre, calcium, vitamin C), dietary intake of nutrients related to brain development (PUFAs, vitamin B_12_, folate), serum concentrations of Hb, vitamin D, PUFAs and C‐reactive protein at the first and third trimester of pregnancy and serum concentrations of RBC folate and vitamin B_12_ at the first trimester of pregnancy. Maternal emotional status, including postpartum depression, were also considered potential confounding factors, as children's behavioural assessments by the CBCL1½‐5 and BRIEF‐P rely on parental responses. Consequently, the mother's emotional state could significantly affect her perceptions and evaluations of her child's behaviour. Incorporating maternal emotional status and postpartum depression as confounders aimed to mitigate this potential bias. As for the child's covariates, the following were included: gestational age, sex, head circumference at birth, energy intake and dietary intake of iron, PUFAs, vitamin B_12_ and folate at 4 years of age. Child IQ was also included as a confounder aiming to isolate and highlight the genuine influence of the intervention on behaviour, thereby ensuring that any observed changes were not due to cognitive variations.

Statistical analyses were done using SPSS software (version 27.0 for Windows; SPSS Inc.).

### 2.4 Ethics statement

The study was designed in agreement with the Declaration of Helsinki/Tokyo. All procedures involving human subjects were approved by the Clinical Research Ethics Committee of the Jordi Gol University Institute for Primary Care Research (Institut d'Investigació en Atenció Primària), the Pere Virgili Health Research Institute (Institut d'Investigació Sanitària Pere Virgili) and of the Spanish Agency for Medicines and Medical Devices (Agencia Española del Medicamento y Productos Sanitarios). Signed informed consent was obtained from all women participating in the study. The quality of the present population‐based randomized controlled trial has been assessed by the Consolidated Standards of Reporting Trials.

## 3 RESULTS

The current analyses were based on a sub‐sample of the ECLIPSES study of 230 mother‐child pairs from which the assessment of children's behaviour and the risk of having behavioural problems at 4 years of age were available (Figure 1). Table 1 shows the main characteristics of the participants included and not included in the present sample with no statistically significant differences between them. The maternal characteristics of the participants included in the present analyses were also described according to their baseline iron status and dose of iron supplementation in Supporting Information S1: Table 1.

**Table 1:** Maternal characteristics of participants included and not included in the present analyses.

	Included (*n* = 230)	Not included (*n* = 561)	*p*
Baseline			
Age, years	32 (26–38)	31 (24–38)	0.098
Parity, yes	55.8	57.1	0.410
Pregnancy planning, yes	84.0	82.3	0.201
Body mass index			0.881
Underweight	1.9	1.7	
Normal weight	56.4	58.5	
Overweight	27.9	25.4	
Obesity	13.8	14.4	
Smoking, yes	16.9	18.4	0.770
Family socioeconomic status			0.235
High	22.9	18.7	
Middle	66.5	67.4	
Low	10.7	13.9	
*HFE* gene mutation, yes	32.3	33.6	0.732
Whole pregnancy			
Physical activity			0.419
Low	26.7	22.6	
Moderate	68.1	70.1	
High	5.2	7.3	
Anxiety^a^			
Trait	14.49 (8.52)	13.55 (8.06)	0.065
State	15.15 (7.31)	14.94 (7.65)	0.158
After delivery			
Postpartum depression^b^	6.82 (4.93)	6.87 (4.98)	0.925

*Note*: Data are expressed in mean (SD) for continuous normally distributed variables, median (Q1–Q3) for continuous non‐normally distributed variables, and % [n] for categorical variables.

^a^ Measured by State–Trait Anxiety Inventory. The score ranges from 0 to 60 points.

^b^ Measured by the Edinburgh Postnatal Depression Scale. The score ranges from 0 to 30 points.

Scores obtained by children aged 4 years on the CBCL1½‐5 and TRF1½‐5 for the intervention (80 or 20 mg/day iron) and control group (40 mg/day iron) according to the mother's baseline iron stores in each Stratum were shown in Supporting Information S1: Tables 2 and 3, respectively. In analyses, some differences were already found between the control and intervention groups, and in the effect of iron doses according to maternal iron stores at baseline. Multivariable analyses provided estimates of the effect of different doses of prenatal iron supplementation on children's behavioural problems. First, no difference was found in the CBCL1½‐5, TRF and BRIEF‐P scores when comparing the intervention and control group in each Stratum by the mother's baseline Hb levels without considering their baseline iron stores (data not shown). However, the analyses stratified by the women's baseline iron stores, classified according to their SF concentrations, unveiled various associations between different doses of prenatal iron supplementation on the child's psychopathology. The tested iron dose (80 mg/day) in Stratum 1 compared with 40 mg/day was associated with lower scores in all the CBCL1½‐5 scales, including internalizing, externalizing, and total problems as well as the DSM scales when women started pregnancy with ID, while it was associated with higher scores in most of the CBCL1½‐5 scales except for depressive problems and attention/hyperactivity problems when women started pregnancy with SF > 65 µg/L. In Stratum 2, the tested iron dose (20 mg/day) compared with 40 mg/day led to lower scores in some of the CBCL1½‐5 scales only in the group of children whose mothers showed baseline SF > 65 µg/L, as follows: externalizing problems (*β*: −9.26, 95% confidence interval [95% CI]: −9.91, −8.61), total problems (*β*: −6.31, 95% CI: −6.35, −6.28), anxiety problems (*β*: −3.18, 95% CI: −5.88, −0.49) and autism spectrum problems (*β*: −5.72, 95% CI: −5.86, −5.58) (Table 2). Although the primary scale “internalizing problems” did not get affected by the intervention in Stratum 2, two items that compose that scale showed lower scores in children from mothers with initial SF > 65 µg/L receiving 20 mg/day instead of 40 mg/day of iron: emotional reactivity (*β*: −4.70, 95% CI: −5.16, −4.25) and withdrawal (*β*: −3.43, 95% CI: −4.93, −1.92) (data not shown). The results obtained from the information reported by the teachers through the TRF1½‐5, although less than that obtained from the parents as expected, reinforced those of the CBCL1½‐5 (Table 3).

**Table 2:** Effect of prenatal iron supplementation by Strata and maternal iron stores in early pregnancy on behavioural problems at 4 years of age, measured by CBCL1½‐5.

	SF < 15 µg/L (*n* = 21)			SF 15–65 µg/L (*n* = 104)			SF > 65 µg/L (*n* = 26)		
Stratum 1 (0: 40 mg/day, 1: 80 mg/day)	*β*	95% CI	*p*	*β*	95% CI	*p*	*β*	95% CI	*p*
Internalizing problems	**−9.61**	**−12.88, −2.34**	**0.001**	−1.02	−9.97, 7.94	0.810	**6.53**	**4.15, 10.83**	**0.010**
Externalizing problems	**−3.44**	**−6.67, −1.80**	**0.008**	6.85	−1.11, 14.80	0.086	**1.61**	**0.74, 6.53**	**0.038**
Total problems	**−6.30**	**−9.15, −3.46**	**0.009**	2.84	−4.51, 10.20	0.418	**1.10**	**0.15, 5.94**	**0.046**
DSM Scales									
Depressive problems	**−6.54**	**−10.75, −3.58**	**0.029**	4.48	−0.24, 9.19	0.061	10.00	−15.10, 35.10	0.294
Anxiety problems	**−3.25**	**−8.24, −1.13**	**0.015**	2.44	−3.63, 8.51	0.403	**5.35**	**2.20, 9.50**	**0.001**
Autism spectrum problems	**−5.26**	**−7.38, −1.04**	**0.045**	−0.19	−8.27, 7.89	0.961	**1.17**	**0.37, 15.97**	**0.047**
Attention‐deficit/hyperactivity problems	**−3.90**	**−8.51, −1.29**	**0.037**	**7.78**	**3.35, 10.22**	**<0.001**	9.67	−9.65, 28.99	0.210
Oppositional defiant problems	**−3.67**	**−9.40, −0.93**	**0.043**	**2.03**	**0.22, 3.84**	**0.030**	**2.69**	**0.11, 5.26**	**0.046**

	SF < 15 µg/L (*n* = 10)			SF 15–65 µg/L (*n* = 54)			SF > 65 µg/L (*n* = 15)		
Stratum 2 (0: 40 mg/day, 1: 20 mg/day)	*β*	95% CI	*p*	*β*	95% CI	*p*	*β*	95% CI	*p*
Internalizing problems	3.51	−2.54, 5.49	0.548	0.09	−13.29, 13.47	0.989	−2.50	−12.78, 7.80	0.496
Externalizing problems	1.06	−2.61, 4.26	0.749	3.64	−8.34, 15.61	0.527	**−9.26**	**−9.91, −8.61**	**0.003**
Total problems	−4.16	−9.32, 8.54	0.641	−0.78	−13.12, 11.57	0.895	**−6.31**	**−6.35, −6.28**	**<0.001**
DSM Scales									
Depressive problems	7.15	−3.25, 11.73	0.594	2.53	−5.66, 10.72	0.521	−4.14	−12.07, 3.78	0.195
Anxiety problems	2.83	−5.17, 6.25	0.684	−5.58	−9.23, 8.06	0.888	**−3.18**	**−5.88, −0.49**	**0.037**
Autism spectrum problems	3.42	−4.84, 6.45	0.491	−4.21	−11.02, 2.59	0.207	**−5.72**	**−5.86, −5.58**	**0.001**
Attention‐deficit/hyperactivity problems	4.16	−6.15, 7.65	0.562	2.83	−7.35, 13.01	0.564	−1.76	−14.66, 11.15	0.694
Oppositional defiant problems	6.94	−2.96, 12.68	0.314	1.02	−6.71, 8.74	0.783	−2.99	−16.66, 10.67	0.536

*Note*: Statistically significant associations are highlighted in bold. Models adjusted by family socioeconomic status (low, middle, high), smoking at recruitment (yes/no), maternal emotional status during pregnancy, postpartum depression, parental IQ approximation, maternal diet (energy, fibre, iron, PUFAs, calcium, vitamin C, vitamin B_12_, folate), serum concentrations of Hb, vitamin D, PUFAs, and C‐reactive protein at the first and third trimester of pregnancy, RBC folate and vitamin B_12_ concentrations at the first trimester of pregnancy, and SF concentrations at the third trimester of pregnancy, gestational age, child sex, children's IQ, head circumference at birth and child diet at 4 years of age (energy, iron, PUFAs, vitamin B_12_, folate).

Abbreviations: CBCL½‐5, Child Behaviour Checklist for ages 1.5–5 years; CI, confidence interval; IQ, intelligence quotient; PUFA, polyunsaturated fatty acid; RBC, red blood cell.

**Table 3:** Effect of prenatal iron supplementation by Strata and maternal iron stores in early pregnancy on behavioural problems at 4 years of age, measured by TRF1½‐5.

	SF < 15 µg/L (*n* = 18)			SF 15–65 µg/L (*n* = 87)			SF > 65 µg/L (*n* = 18)		
Stratum 1 (0: 40 mg/day, 1: 80 mg/day)	*β*	95% CI	*p*	*β*	95% CI	*p*	*β*	95% CI	*p*
Internalizing problems	−7.68	−18.67, 3.30	0.071	8.24	−0.27, 16.74	0.057	**9.31**	**6.95, 15.66**	**0.019**
Externalizing problems	**−4.92**	**−18.57, −1.27**	**0.003**	−4.38	−12.86, 4.11	0.287	**5.95**	**3.54, 8.36**	**0.020**
Total problems	−3.04	−18.72, 12.64	0.246	5.71	−1.81, 13.24	0.126	**9.46**	**7.07, 11.85**	**0.013**
**DSM Scales**									
Depressive problems	−8.33	−31.28, 14.61	0.259	3.58	−1.20, 8.36	0.131	**3.02**	**2.51, 3.53**	**0.009**
Anxiety problems	**−7.70**	**−13.57, −1.83**	**0.038**	4.29	−1.54, 10.11	0.138	4.67	−14.51, 23.84	0.495
Autism spectrum problems	1.33	−10.14, 12.81	0.667	3.61	−1.25, 8.47	0.130	2.67	−4.58, 9.91	0.326
Attention‐deficit/hyperactivity problems	3.51	−3.21, 10.24	0.095	−1.32	−6.19, 3.54	0.567	3.24	−0.65, 7.12	0.070
Oppositional defiant problems	**−7.54**	**−20.34, −2.74**	**0.038**	−2.40	−6.98, 2.19	0.281	−3.38	−8.06, 1.30	0.190

	SF < 15 µg/L (*n* = 8)			SF 15–65 µg/L (*n* = 47)			SF > 65 µg/L (*n* = 12)		
Stratum 2 (0: 40 mg/day, 1: 20 mg/day)	*β*	95% CI	*p*	*β*	95% CI	*p*	*β*	95% CI	*p*
Internalizing problems	−4.12	−9.54, 7.52	0.549	−3.00	−8.33, 2.32	0.237	**−7.41**	**−10.02, −4.80**	**0.018**
Externalizing problems	−8.41	−12.63, 0.97	0.080	−0.84	−5.98, 4.29	0.719	1.17	−26.04, 28.38	0.900
Total problems	−6.87	−10.39, 2.64	0.105	−1.59	−8.97, 5.78	0.644	0.73	−7.11, 8.56	0.729
**DSM Scales**									
Depressive problems	−1.59	−3.49, 2.17	0.491	−0.24	−5.76, 5.29	0.928	2.33	−23.69, 28.35	0.974
Anxiety problems	0.51	−1.25, 2.16	0.812	0.60	−1.38, 2.57	0.512	**−8.79**	**−10.30, −7.27**	**0.009**
Autism spectrum problems	−1.54	−3.81, 2.98	0.694	−3.77	−10.88, 3.34	0.273	**−1.64**	**−1.72, −1.57**	**0.002**
Attention‐deficit/hyperactivity problems	−1.62	−3.62, 2.63	0.259	−4.57	−16.40, 7.26	0.421	1.50	−24.83, 27.83	0.868
Oppositional defiant problems	−3.45	−6.41, 3.10	0.463	−6.05	−8.63, −3.47	<0.001	**−3.80**	**−5.53, −2.07**	**0.023**

*Note*: Statistically significant associations are highlighted in bold. Models adjusted by family socioeconomic status (low, middle, high), smoking at recruitment (yes/no), maternal emotional status during pregnancy, postpartum depression, parental IQ approximation, maternal diet (energy, fibre, iron, PUFAs, calcium, vitamin C, vitamin B_12_, folate), serum concentrations of Hb, vitamin D, PUFAs, and C‐reactive protein at the first and third trimester of pregnancy, RBC folate and vitamin B12 concentrations at the first trimester of pregnancy, and SF concentrations at the third trimester of pregnancy, gestational age, child sex, children's IQ, head circumference at birth and child diet at 4 years of age (energy, iron, PUFAs, vitamin B_12_, folate).

Abbreviations: CI, confidence interval; IQ, intelligence quotient; PUFA, polyunsaturated fatty acid; RBC, red blood cell; SF, serum ferritin; TRF1½‐5, Teacher's Report Form for ages 1.5–5 years.

As for the executive functioning of children at 4 years of age, the tested iron dose (80 mg/day) in Stratum 1 compared with 40 mg/day was associated with lower scores in all the BRIEF‐P items except for flexibility when women started pregnancy with SF < 15 µg/L, whereas it was associated with higher scores in flexibility, emotional control, plan/organize and global executive index when women started pregnancy with SF > 65 µg/L. In Stratum 2, the tested iron dose (20 mg/day) compared with 40 mg/day led to lower scores in emotional control, working memory and behavioural regulation index only in the group of children whose mothers showed baseline SF > 65 µg/L (Table 4).

**Table 4:** Effect of prenatal iron supplementation by Strata and maternal iron stores in early pregnancy on executive functions at 4 years of age, measured by the BRIEF‐P.

	SF < 15 µg/L (*n* = 21)			SF 15–65 µg/L (*n* = 104)			SF > 65 µg/L (*n* = 26)		
Stratum 1 (0: 40 mg/day, 1: 80 mg/day)	*β*	95% CI	*p*	*β*	95% CI	*p*	*β*	95% CI	*p*
Inhibition	**−6.26**	**−7.98, −4.54**	**0.014**	1.16	−4.17, 6.50	0.651	−0.46	−3.47, 2.56	0.583
Flexibility	−7.00	−59.34, 45.34	0.623	0.81	−7.90, 9.52	0.847	**6.50**	**1.54, 11.46**	**0.022**
Emotional control	**−4.51**	**−7.18, −2.84**	**0.015**	**5.48**	**1.81, 9.15**	**0.006**	**9.64**	**13.40, 5.87**	**0.001**
Working memory	**−5.40**	**−10.43, −3.37**	**<0.001**	2.24	−4.68, 9.15	0.504	4.25	−9.18, 17.68	0.429
Plan/organize	**−6.59**	**−12.95, −3.01**	**0.030**	−0.17	−7.97, 7.64	0.964	**8.29**	**7.73, 8.86**	**0.003**
Indices									
Behavioural Regulation Index	**−7.11**	**−9.69, −4.54**	**0.018**	1.84	−2.68, 6.66	0.431	−1.00	−11.62, 9.62	0.807
Flexibility Index	−12.00	−54.38, 30.38	0.347	2.34	−4.16, 8.83	0.458	4.85	−0.08, 9.77	0.052
Metacognition Index	**−8.37**	**−14.23, −6.51**	**0.021**	1.22	−5.93, 8.36	0.723	1.25	−16.87, 19.37	0.857
Global Executive Index	**−7.51**	**−10.49, −2.53**	**0.012**	1.88	−3.67, 7.73	0.484	**6.21**	**3.12, 9.31**	**0.020**

	SF < 15 µg/L (*n* = 10)			SF 15‐65 µg/L (*n* = 54)			SF > 65 µg/L (*n* = 15)		
Stratum 2 (0: 40 mg/day, 1: 20 mg/day)	*β*	95% CI	*p*	*β*	95% CI	*p*	*β*	95% CI	*p*
Inhibition	2.35	−4.85, 5.30	0.258	4.23	−4.47, 12.93	0.311	−2.31	−13.54, 8.92	0.599
Flexibility	1.71	−3.14, 2.99	0.146	0.36	−9.63, 10.34	0.940	1.10	−12.70, 14.90	0.846
Emotional control	0.95	−2.51, 4.15	0.365	1.39	−8.60, 11.37	0.770	**−7.69**	**−15.73, −5.65**	**0.007**
Working memory	−1.24	−4.20, 3.21	0.314	−0.19	−13.42, 13.03	0.975	**−8.90**	**−17.70, −0.10**	**0.048**
Plan/organize	−2.36	−5.16, 1.78	0.152	4.96	−7.92, 17.83	0.425	2.80	−14.80, 20.40	0.699
Indices									
Behavioural Regulation Index	3.87	−1.94, 6.57	0.225	2.50	−8.05, 13.04	0.619	**−4.43**	**−6.57, −2.29**	**0.012**
Flexibility Index	1.60	−2.63, 5.40	0.394	−0.76	−12.21, 10.68	0.889	5.88	−2.02, 13.78	0.108
Metacognition Index	−3.28	−7.15, 2.38	0.184	1.97	−11.67, 15.62	0.762	−4.90	−16.35, 6.55	0.321
Global Executive Index	−0.15	−4.68, 3.95	0.471	0.32	−13.44, 14.08	0.961	−0.93	−4.26, 2.40	0.440

*Note*: Statistically significant associations are highlighted in bold. Models adjusted by family socioeconomic status (low, middle, high), smoking at recruitment (yes/no), maternal emotional status during pregnancy, postpartum depression, parental IQ approximation, maternal diet (energy, fibre, iron, PUFAs, calcium, vitamin C, vitamin B_12_, folate), serum concentrations of Hb, vitamin D, PUFAs, and C‐reactive protein at the first and third trimester of pregnancy, RBC folate and vitamin B_12_ concentrations at the first trimester of pregnancy, and SF concentrations at the third trimester of pregnancy, gestational age, child sex, children's IQ, head circumference at birth and child diet at 4 years of age (energy, iron, PUFAs, vitamin B_12_, folate).

Abbreviations: BRIEF‐P, Behaviour Rating Inventory of Executive Function‐Preschool Version; IQ, intelligence quotient; PUFA, polyunsaturated fatty acid; RBC, red blood cell; SF, serum ferritin.

## 4 DISCUSSION

The present study found that adapting prenatal iron supplementation in non‐anaemic women according to their Hb levels and iron reserves in early pregnancy would reduce behavioural problems in children aged 4 years. We obtained information from both parents and teachers and observed that the relationship between iron supplementation adjusted to maternal baseline SF levels and children's behavioural problems was more evident when considering information reported by parents. Some studies have previously shown differences between the data reported by different informants, with parents more frequently rating their children higher than teachers on behavioural problems and hyperactivity (Sans et al., 2021), as well as on other psychological problems (Martinsone et al., 2022).

To the best of our knowledge, this is the first study that considers together Hb level and iron stores at the beginning of pregnancy to evaluate the effect of different doses of prenatal iron supplements on the child's behavioural problems, which makes it difficult to compare the current results. Previous studies evaluating the effects of routine prenatal iron supplementation with a single dose of iron failed to provide clear evidence of benefits. As it is well known that both ID and excess during pregnancy can have long‐term consequences on the child's neuropsychological development and increase the risk of behavioural problems by being detrimental to the development and maturation of the fetal brain, one could argue that having administered a single dose to all women in those studies may have been insufficient or excessive, depending on the case, to observe positive effects or even to report, by contrary, adverse effects after prenatal supplementation.

It is worth mentioning that as anaemia was an exclusion criterion for participation in the study, none of the participants was anaemic at the beginning of pregnancy, but some of them showed ID at that time. Further, this point is relevant in the present study, because the differences found in the effect of prenatal iron doses on children's behavioural problems depended on women's iron stores at the beginning of pregnancy beyond their Hb levels. Specifically, prenatal administration of high‐dose iron (80 mg/day) in women with normal Hb concentration improved, according to information reported by parents, most of the executive functions and reduced all the behavioural problems in their 4‐year‐old children when the women had baseline ID, whereas it resulted in worse scores for some items about executive functioning, as well as for internalizing, externalizing and total problems, and most DSM scales, specifically autism spectrum problems in the children of those who began pregnancy with normal‐high iron stores (SF ≥ 65 µg/L). This is consistent with previous results from our own study regarding different scales of child cognitive development (Iglesias‐Vázquez et al., 2023), including working memory which is a component of executive functioning and is associated with ADHD. This observation indicates that, in the first case, an iron dose higher than that usually prescribed in Spain (40 mg/day) compensated for the low iron stores that could have undermined the children's behavioural development at 4 years of age, although the Hb levels did not indicate the presence of anaemia, which until now would not have justified the prescription of higher prenatal doses of iron in routine clinical practice. On the other hand, the results suggest that having received a high dose of iron could have led to an excess of iron in the second case when the women started the pregnancy with normal‐high iron stores, which translated into poorer behavioural development of their children. These findings highlight that not only is the prevention of ID important, but the prevention of possible iron excess leads to better emotional and behavioural development of the child. The results in Stratum 2 supported this argument, with those with SF ≥ 65 µg/L showing better performance in emotional control, working memory and behaviour regulation, externalizing and total problems, as well as the DSM scales anxiety and autism spectrum problems in children whose mothers had received low‐dose iron supplementation (20 mg/day) prenatally, again indicating the need for prevention of iron excess when aiming to improve the child behavioural development.

That a high prenatal iron dose improves the behaviour and executive functioning and reduces behavioural problems of children when women entered the pregnancy with ID was an expected finding. Indeed, there is abundant evidence in the literature that low iron status during pregnancy is associated with an increased risk of a wide range of neurodevelopmental disorders in children (Gaillard et al., 2014; Georgieff, 2008; Hernández‐Martínez et al., 2011; Iglesias et al., 2017; Radlowski & Johnson, 2013). Along the same line, higher maternal SF levels, the use of prenatal iron supplementation, and higher overall iron intake generally have shown a protective effect against such problems (Arija et al., 2019; Díaz‐López et al., 2022; Santa‐Marina et al., 2020; Schmidt et al., 2014). In contrast, although some studies reported no improvement in the outcomes studied in relation to child cognitive or behavioural development following prenatal iron supplementation (Jayasinghe et al., 2018; Parsons et al., 2008; Zhou et al., 2006), the detrimental effect of high‐dose supplementation observed in the present study in children of mothers who started pregnancy with full iron stores was not so clear until now. One explanation for this finding is that giving high doses of prenatal iron to women who do not really need it could lead to excess iron, causing oxidative stress and even iron deposits that, in turn, could impair foetal brain development and lead to a poorer behavioural and psychological performance in infancy (Lavezzi et al., 2011; Zerem et al., 2021). Recent research has noted that epigenetics may also play a role in the association between maternal iron status and children's behavioural development by differential DNA methylation (Taeubert et al., 2022). As the authors explained, oxidative stress would be the key behind this physiological process as it leads to a reduction in enzymatic activity important during DNA demethylation (Niu et al., 2015).

### 4.1 Strengths and limitations

The main strengths of the present study were (1) the original study was a population‐based triple‐blind randomized controlled trial, which is a very robust design; (2) comprehensive data on sociodemographic, clinical and lifestyle information of the mothers were recorded; and (3) the assessment of children's behaviour was conducted comprehensively using reliable and internationally recognized tests. Although this study has clinical implications and will be helpful for future research in this area, it is important to keep in mind some limitations. The main limitation was the small sample size of the subgroups analysed because of high losses to follow‐up which, despite being common in population‐based intervention studies and even more so when prolonged follow‐up is required, could have weakened the statistical power of the analyses. In addition, unmeasured or unknown risk factors could have resulted in some residual confounding even after adjusting for known potential confounders. Therefore, drawing absolute conclusions may be premature and further research is warranted.

## 5 CONCLUSIONS

Adjusting prenatal iron supplementation to maternal baseline iron stores even in nonanaemic women improves executive functioning and reduces behavioural problems in children aged 4 years. The best‐performing children were those whose mothers prenatally received 80 mg of iron daily when they started pregnancy with normal Hb concentrations but ID, those whose mothers received 40 mg of iron daily when they started pregnancy with normal Hb levels and good iron reserves, and those whose mothers received 20 mg of iron daily when they started pregnancy with normal‐high Hb concentrations and SF > 65 µg/L. Therefore, our data experimentally support that prenatal high‐dose iron supplementation is beneficial for women with ID without anaemia in early pregnancy and for their children, whereas it appears to lead to iron excess iron in those who enter gestation with full iron stores, which may lead to negative mental health outcomes in children later in life. The latter as well as their offspring would benefit from the use of prenatal iron supplements in low doses, as reflected in behavioural developmental outcomes in children. the present results provide valuable information for obstetricians and midwives in pregnancy planning services. Given that SF concentration is not commonly measured in clinical practice, key information is being lost in deciding the best dose of prenatal iron supplementation for each woman. For this reason, routine measuring of SF in addition to Hb concentration from early pregnancy would be advisable to be able to help women to reach and maintain good iron status along gestation, which will result in better behavioural development and fewer behavioural problems for their children. Further studies would be useful to replicate and verify the present findings.

## AUTHOR CONTRIBUTIONS

Lucía Iglesias‐Vázquez, Josefa Canals, Carmen Hernández‐Martínez, Núria Voltas and Victoria Arija performed the research. Josefa Canals and Victoria Arija designed the research study. Lucía Iglesias‐Vázquez analysed the data and wrote the paper. All the authors revised and agreed to the final version of the paper.

## CONFLICT OF INTEREST STATEMENT

The authors declare no conflict of interest.

## TRIAL REGISTRATION

The ECLIPSES study was registered at www.clinicaltrialsregister.eu as EudraCT number 2012‐005480‐28 on 21 May 2013.

## Supporting information

Supporting Information.

## Data Availability

The data that support the findings of this study are available from the corresponding author upon reasonable request.
