# Supplementary material for: Prenatal iron supplementation adjusted to maternal iron stores reduces behavioural problems in 4‐year‐old children
Source: Matern Child Nutr. 2023 Dec 2;20(1):e13595. doi: 10.1111/mcn.13595 (PMC10750013; doi:10.1111/mcn.13595)
Supplement: Supplementary file 1 — Supporting Information. [file MCN-20-e13595-s001.docx]

| **Appendix Table 1. Maternal characteristics according to their baseline iron status and dose of iron supplementation.** | | | | | | | | | | | | | | | | | | |
| --- | --- | --- | --- | --- | --- | --- | --- | --- | --- | --- | --- | --- | --- | --- | --- | --- | --- | --- |
|  |  | ***Stratum* 1** | | | | | | | |  | ***Stratum* 2** | | | | | | | |
|  |  | **SF<15 µg/L** | |  | **SF 15-65 µg/L** | |  | **SF>65 µg/L** | |  | **SF<15 µg/L** | |  | **SF 15-65 µg/L** | |  | **SF>65 µg/L** | |
|  |  | **C** | **I** |  | **C** | **I** |  | **C** | **I** |  | **C** | **I** |  | **C** | **I** |  | **C** | **I** |
|  |  | **n=16** | **n=13** |  | **n=63** | **n=61** |  | **n=11** | **n=18** |  | **n=8** | **n=5** |  | **n=39** | **n=38** |  | **n=9** | **n=10** |
| *Baseline* |  |  |  |  |  |  |  |  |  |  |  |  |  |  |  |  |  |  |
| Age, years |  | 32 ± 7 | 33 ± 5 |  | 32 ± 6 | 32 ± 6 |  | 30,5 ± 8 | 31 ± 6 |  | 32,5 ± 11 | 32 ± 7 |  | 33 ± 8 | 33 ± 6 |  | 29 ± 4 | 29 ± 6 |
| Parity, yes |  | 81.3 [13] | 85.7 [11] |  | 56.5 [36] | 52.1 [32] |  | 58.3 [6] | 52.6 [9] |  | 87.5 [7] | 60.0 [3] |  | 47.5 [18.5] | 56.1 [2] |  | 36.4 [3] | 27.3 [3] |
| Pregnancy planning, yes |  | 75.0 [12] | 85.7 [11] |  | 88.4 [56] | 74.6 [46] |  | 100 [11] | 89.5 [16] |  | 87.5 [7] | 100 [5] |  | 77.5 [30] | 90.2 [34] |  | 100 [9] | 72.7 [7] |
| Body mass index |  |  |  |  |  |  |  |  |  |  |  |  |  |  |  |  |  |  |
| Underweight |  | 0 [0] | 7.1 [1] |  | 2.9 [2] | 0 [0] |  | 0 [0] | 5.3 [1] |  | 12.5 [1] | 0 [0] |  | 0 [0] | 0 [0] |  | 0 [0] | 9.1 [1] |
| Normal weight |  | 87.5 [14] | 57.1 [7] |  | 59.4 [37] | 47.9 [29] |  | 58.3 [6] | 73.7 [13] |  | 50.0 [4] | 40.0 [2] |  | 47.5 [19] | 61.0 [23] |  | 36.4 [3] | 54.5 [5] |
| Overweight |  | 6.3 [1] | 14.3 [2] |  | 21.7 [14] | 39.4 [24] |  | 41.7 [5] | 10.5 [2] |  | 25.0 [2] | 20.0 [1] |  | 46.2 [18] | 22.0 [9] |  | 36.4 [3] | 18.2 [2] |
| Obesity |  | 6.3 [1] | 21.4 [3] |  | 15.9 [10] | 12.7 [8] |  | 0 [0] | 10.5 [2] |  | 12.5 [1] | 40.0 [2] |  | 5.0 [2] | 17.1 [6] |  | 36.4 [3] | 18.2 [2] |
| Smoking, yes |  | 6.3 [1] | 7.1 [1] |  | 14.5 [9] | 19.7 [12] |  | 8.3 [1] | 15.8 [3] |  | 12.5 [1] | 20.0 [1] |  | 25 [10] | 17.1 [6] |  | 27.3 [2] | 18.2 [2] |
| Family SES |  |  |  |  |  |  |  |  |  |  |  |  |  |  |  |  |  |  |
| High |  | 18.8 [3] | 14.3 [2] |  | 26.1 [16] | 21.1 [13] |  | 33.3 [3] | 26.3 [5] |  | 25.0 [2] | 40.0 [2] |  | 27.5 [11] | 14.6 [5] |  | 18.2 [2] | 27.3 [3] |
| Middle |  | 75.0 [12] | 71.4 [9] |  | 68.1 [43] | 62 [38] |  | 50.0 [6] | 57.9 [10] |  | 75.0 [6] | 60.0 [3] |  | 70.0 [27] | 70.7 [28] |  | 66.7 [6] | 63.6 [6] |
| Low |  | 6.3 [1] | 14.3 [2] |  | 5.8 [4] | 16.9 [10] |  | 16.7 [2] | 15.8 [3] |  | 0 [0] | 0 [0] |  | 2.5 [1] | 14.6 [5] |  | 9.1 [1] | 9.1 [1] |
| *HFE* gene mutation, yes |  | 50.0 [8] | 50.0 [7] |  | 50.0 [32] | 50.0 [31] |  | 36.4 [4] | 31.3 [6] |  | 57.1 [5] | 40.0 [2] |  | 41.4 [16] | 24.3 [9] |  | 44.4 [4] | 33.3 [3] |
| Parental IQ approximation |  | 8.40  (4.32) | 9.62  (3.66) |  | 9.78 (3.30) | 8.45  (3.09) |  | 9.83  (4.30) | 8.67  (3.05) |  | 9.75  (5.39) | 8.50  (3.42) |  | 9.28  (3.39) | 8.50  (3.73) |  | 7.30  (2.95) | 9.75  (5.63) |
| *Whole pregnancy* |  |  |  |  |  |  |  |  |  |  |  |  |  |  |  |  |  |  |
| Physical activity |  |  |  |  |  |  |  |  |  |  |  |  |  |  |  |  |  |  |
| Low |  | 26.7 [4] | 23.1 [3] |  | 27.6 [17] | 22.2 [14] |  | 20.0 [2] | 25.1 [5] |  | 25.0 [2] | 40.0 [2] |  | 34.4 [13] | 28.1 [11] |  | 44.4 [4] | 30.0 [3] |
| Moderate |  | 73.3 [12] | 76.9 [10] |  | 69 [43] | 73.0 [45] |  | 50.0 [6] | 75.0 [13] |  | 62.5 [5] | 60.0 [3] |  | 59.4 [23] | 65.6 [25] |  | 55.5 [5] | 70.0 [7] |
| High |  | 0 [0] | 0 [0] |  | 3.4 [3] | 4.8 [3] |  | 30.0 [3] | 0 [0] |  | 12.5 [1] | 0 [0] |  | 6.3 [3] | 6.3 [2] |  | 0 [0] | 0 [0] |
| Anxiety^*^ |  |  |  |  |  |  |  |  |  |  |  |  |  |  |  |  |  |  |
| Trait |  | 17.45 (8.84) | 15.48  (10.57) |  | 12.47 (7.54) | 16.89  (8.74) |  | 15.69 (7.70) | 12.02  (8.95) |  | 9.72  (6.32) | 15.15  (7.12) |  | 14.40  (8.00) | 13.62  (9.59) |  | 13.11 (4.16) | 16.55  (10.43) |
| State |  | 17.43 (7.80) | 16.57  (7.24) |  | 13.44 (7.06) | 16.95  (7.79) |  | 15.54 (5.95) | 12.81  (6.99) |  | 11.78 (4.48) | 18.85  (6.52) |  | 15.63  (7.28) | 13.41  (7.09) |  | 15.14 (5.18) | 17.50  (8.71) |
| *After delivery* |  |  |  |  |  |  |  |  |  |  |  |  |  |  |  |  |  |  |
| Postpartum depression^†^ |  | 5.67  (5.55) | 6.67  (6.18) |  | 5.81 (3.92) | 8.51  (5.51) |  | 6.20  (4.05) | 6.40  (3.03) |  | 2.40  (1.95) | 5.80  (2.49) |  | 7.38  (4.64) | 7.80  (6.42) |  | 5.17  (5.31) | 6.56  (4.25) |
| Data are expressed in mean (SD) for continuous normally distributed variables, median ± interquartile range for continuous non-normally distributed variables, and % [n] for categorical variables. | | | | | | | | | | | | | | | | | | |
| C, control; I, intervention; SF, serum ferritin; SES, socioeconomic status. | | | | | | | | | | | | | | | | | | |
| The control group always received 40 mg/d of iron while the intervention group received 80 mg/d of iron in *Stratum* 1 and 20 mg/d of iron in *Stratum* 2. | | | | | | | | | | | | | | | | | | |
| ^*^Measured by STAI questionnaire. The score ranges from 0 to 60 points. | | | | | | | | | | | | | | | | | | |
| ^†^Measured by Edinburg questionnaire. The score ranges from 0 to 30 points. | | | | | | | | | | | | | | | | | | |

| **Appendix Table 2. CBCL1½-5 scores at 4y of age by different daily doses of prenatal iron supplementation by *Strata* and maternal baseline iron stores (n=230).** | | | | | | | | | | | | | | | | | |  |
| --- | --- | --- | --- | --- | --- | --- | --- | --- | --- | --- | --- | --- | --- | --- | --- | --- | --- | --- |
| ***Stratum* 1 (Hb 110-130 g/L)** |  |  |  |  |  |  |  |  |  |  |  |  |  |  |  |  |  | |
|  | **SF<15 µg/L** | | | | |  | **SF 15-65 µg/L** | | | | |  | **SF>65 µg/L** | | | | | |
|  | **40 mg/d**  **(n=11)** | | **80 mg/d**  **(n=10)** | | **p** |  | **40 mg/d**  **(n=54)** | | **80 mg/d**  **(n=50)** | | **p** |  | **40 mg/d**  **(n=11)** | | **80 mg/d**  **(n=15)** | | **p** | |
|  | **Mean** | **SD** | **Mean** | **SD** |  |  | **Mean** | **SD** | **Mean** | **SD** |  |  | **Mean** | **SD** | **Mean** | **SD** |  |  |
| Internalizing Problems | **57.00** | **15.83** | **51.70** | **11.45** | **0.018^a^** |  | 57.00 | 11.60 | 53.26 | 11.04 | 0.095 |  | **55.53** | **11.36** | **60.67** | **13.60** | **0.033^a^** | |
| Externalizing Problems | **55.18** | **13.78** | **51.50** | **7.25** | **0.045^a^** |  | 52.37 | 9.63 | 55.26 | 10.80 | 0.152 |  | 52.67 | 13.66 | 54.87 | 9.97 | 0.253 | |
| Total Problems | 56.91 | 17.12 | 51.30 | 9.89 | 0.076 |  | 52.76 | 10.59 | 57.00 | 12.10 | 0.060 |  | 56.60 | 12.66 | 57.89 | 17.11 | 0.134 | |
| DSM Scales |  |  |  |  |  |  |  |  |  |  |  |  |  |  |  |  |  | |
| Depressive Problems | **58.82** | **9.15** | **53.40** | **5.23** | **0.017^b^** |  | 55.78 | 6.75 | 57.88 | 7.86 | 0.146 |  | 57.60 | 8.07 | 58.22 | 10.17 | 0.270 | |
| Anxiety Problems | **58.00** | **14.02** | **55.70** | **5.79** | **0.026^a^** |  | 56.83 | 6.97 | 59.24 | 9.18 | 0.138 |  | **56.93** | **6.93** | **59.22** | **7.95** | **0.046^b^** | |
| Autism Spectrum Problems | 59.36 | 9.23 | 55.60 | 6.92 | 0.068 |  | 59.24 | 7.28 | 56.46 | 7.23 | 0.054 |  | 58.67 | 8.15 | 59.78 | 10.18 | 0.171 | |
| Attention-Deficit/Hyperactivity Problems | 59.45 | 9.41 | 53.80 | 5.45 | 0.057 |  | 55.98 | 5.55 | 58.50 | 8.58 | 0.082 |  | 56.78 | 9.71 | 57.67 | 6.03 | 0.783 | |
| Oppositional Defiant Problems | 57.55 | 9.19 | 52.20 | 2.15 | 0.087 |  | 54.31 | 6.10 | 55.48 | 6.87 | 0.362 |  | 54.67 | 6.61 | 55.22 | 6.92 | 0.086 | |
| ***Stratum* 2 (Hb>130 g/L)** |  |  |  |  |  |  |  |  |  |  |  |  |  |  |  |  |  | |
|  | **SF<15 µg/L** | | | | |  | **SF 15-65 µg/L** | | | | |  | **SF>65 µg/L** | | | | | |
|  | **40 mg/d**  **(n=5)** | | **20 mg/d**  **(n=5)** | | **p** |  | **40 mg/d**  **(n=27)** | | **20 mg/d**  **(n=27)** | | **p** |  | **40 mg/d**  **(n=7)** | | **20 mg/d**  **(n=8)** | | **p** | |
|  | **Mean** | **SD** | **Mean** | **SD** |  |  | **Mean** | **SD** | **Mean** | **SD** |  |  | **Mean** | **SD** | **Mean** | **SD** |  |  |
| Internalizing Problems | 42.80 | 23.92 | 47.00 | 13.25 | 0.743 |  | 54.00 | 10.69 | 56.00 | 13.28 | 0.545 |  | 58.14 | 7.36 | 53.75 | 12.44 | 0.429 | |
| Externalizing Problems | 40.20 | 20.04 | 42.40 | 7.40 | 0.824 |  | 52.52 | 12.08 | 54.70 | 10.23 | 0.477 |  | 57.38 | 16.57 | 53.86 | 10.81 | 0.095 | |
| Total Problems | 47.00 | 14.78 | 42.80 | 9.04 | 0.603 |  | 55.63 | 9.59 | 54.96 | 13.70 | 0.837 |  | 57.14 | 9.03 | 54.75 | 14.91 | 0.833 | |
| DSM Scales |  |  |  |  |  |  |  |  |  |  |  |  |  |  |  |  |  | |
| Depressive Problems | 43.20 | 23.56 | 51.60 | 3.05 | 0.472 |  | 57.19 | 7.91 | 57.93 | 7.25 | 0.721 |  | 58.38 | 7.23 | 56.57 | 7.16 | 0.636 | |
| Anxiety Problems | 48.00 | 27.52 | 53.00 | 5.61 | 0.701 |  | 57.96 | 9.46 | 56.63 | 6.05 | 0.540 |  | 57.57 | 6.48 | 55.88 | 6.27 | 0.065 | |
| Autism Spectrum Problems | 47.20 | 25.67 | 54.20 | 5.50 | 0.568 |  | 57.52 | 8.29 | 56.89 | 8.15 | 0.780 |  | 58.43 | 2.70 | 55.88 | 6.40 | 0.059 | |
| Attention-Deficit/Hyperactivity Problems | 45.80 | 24.07 | 50.80 | 0.84 | 0.667 |  | 56.44 | 8.62 | 58.93 | 8.54 | 0.293 |  | 62.00 | 10.41 | 59.88 | 9.64 | 0.688 | |
| Oppositional Defiant Problems | 43.00 | 22.20 | 50.80 | 0.45 | 0.476 |  | 54.37 | 7.55 | 55.44 | 6.71 | 0.583 |  | 58.50 | 9.52 | 54.29 | 4.92 | 0.297 | |
| CBCL, Child Behavior Checklist; SF, serum ferritin; Hb, haemoglobin | | | | | | | | | | | | | | | | | | |
| Statistically significant associations are highlighted in bold. | | | | | | | | | | | | | | | | | | |
| Cohen’s D for assessing effect size was indicated as follows: ^a^Large effect size (>0.8), ^b^Medium effect size (>0.3-0.8), ^c^Low effect size (0.2-0.3). | | | | | | | | | | | | | | | | | | |

| **Appendix Table 3. TRF1½-5 scores at 4y of age by different daily doses of prenatal iron supplementation by *Strata* and maternal baseline iron stores (n=190).** | | | | | | | | | | | | | | | | | | | | |
| --- | --- | --- | --- | --- | --- | --- | --- | --- | --- | --- | --- | --- | --- | --- | --- | --- | --- | --- | --- | --- |
| ***Stratum* 1 (Hb 110-130 g/L)** |  |  |  |  |  |  |  |  |  |  |  | |  |  | |  |  | |  |  |
|  | **SF<15 µg/L** | | | | |  | **SF 15-65 µg/L** | | | | |  | | **SF>65 µg/L** | | | | | | |
|  | **40 mg/d** | | **80 mg/d** | | **p** |  | **40 mg/d** | | **80 mg/d** | | **p** | |  | | **40 mg/d** | | | **80 mg/d** | | **p** |
|  | **(n=9)** | | **(n=9)** | |  |  | **(n=42)** | | **(n=45)** | |  |  |  |  | **(n=11)** | | | **(n=7)** | |  |
|  | **Mean** | **SD** | **Mean** | **SD** |  |  | **Mean** | **SD** | **Mean** | **SD** |  | |  | **Mean** | | **SD** | **Mean** | | **SD** |  |
| Internalizing Problems | 46.00 | 9.77 | 43.11 | 4.54 | 0.438 |  | 47.18 | 8.71 | 51.07 | 10.28 | 0.664 | |  | 46.00 | | 9.96 | 47.86 | | 9.10 | 0.696 |
| Externalizing Problems | 45.56 | 5.98 | 44.67 | 8.43 | 0.800 |  | 50.26 | 8.50 | 49.91 | 10.52 | 0.864 | |  | 48.57 | | 6.50 | 51.45 | | 6.96 | 0.393 |
| Total Problems | 44.89 | 8.51 | 43.67 | 5.07 | 0.716 |  | 47.11 | 10.56 | 49.45 | 9.66 | 0.538 | |  | 48.14 | | 6.54 | 49.91 | | 7.91 | 0.629 |
| DSM Scales |  |  |  |  |  |  |  |  |  |  |  | |  |  | |  |  | |  |  |
| Depressive Problems | 53.11 | 6.86 | 51.22 | 3.67 | 0.477 |  | 52.82 | 4.36 | 54.79 | 7.56 | 0.146 | |  | 53.64 | | 5.56 | 55.29 | | 6.29 | 0.567 |
| Anxiety Problems | 51.22 | 2.54 | 50.89 | 2.57 | 0.588 |  | 53.02 | 4.63 | 54.38 | 5.54 | 0.220 | |  | 53.86 | | 4.91 | 53.82 | | 6.54 | 0.989 |
| Autism Spectrum Problems | 51.22 | 2.22 | 52.11 | 2.89 | 0.475 |  | 43.22 | 5.41 | 54.17 | 7.10 | 0.489 | |  | 53.82 | | 8.59 | 50.86 | | 0.69 | 0.381 |
| Attention-Deficit/Hyperactivity Problems | 51.00 | 2.29 | 52.89 | 5.18 | 0.332 |  | 54.67 | 6.28 | 54.56 | 6.63 | 0.936 | |  | 53.14 | | 3.39 | 54.55 | | 5.20 | 0.538 |
| Oppositional Defiant Problems | 51.00 | 2.65 | 45.32 | 2.12 | 1.000 |  | 53.71 | 5.59 | 52.93 | 4.12 | 0.458 | |  | 53.00 | | 2.89 | 52.64 | | 2.84 | 0.796 |
| ***Stratum* 2 (Hb>130 g/L)** |  |  |  |  |  |  |  |  |  |  |  | |  |  | |  |  | |  |  |
|  | **SF<15 µg/L** | | | | |  | **SF 15-65 µg/L** | | | | |  | | **SF>65 µg/L** | | | | | | |
|  | **40 mg/d** | | **20 mg/d** | | **p** |  | **40 mg/d** | | **20 mg/d** | | **p** | |  | | **40 mg/d** | | | **20 mg/d** | | **p** |
|  | **(n=3)** | | **(n=5)** | |  |  | **(n=25)** | | **(n=22)** | |  |  |  |  | **(n=6)** | | | **(n=6)** | |  |
|  | **Mean** | **SD** | **Mean** | **SD** |  |  | **Mean** | **SD** | **Mean** | **SD** |  | |  | **Mean** | | **SD** | **Mean** | | **SD** |  |
| Internalizing Problems | 47.33 | 11.93 | 43.60 | 10.41 | 0.657 |  | 50.44 | 10.14 | 48.55 | 7.85 | 0.482 | |  | 48.83 | | 10.36 | 45.83 | | 13.14 | 0.670 |
| Externalizing Problems | 57.67 | 10.07 | 43.60 | 8.08 | 0.071 |  | 53.40 | 11.98 | 50.32 | 10.63 | 0.359 | |  | 50.33 | | 9.59 | 51.00 | | 10.47 | 0.911 |
| Total Problems | 52.67 | 11.68 | 41.40 | 10.81 | 0.214 |  | 52.04 | 12.62 | 49.86 | 9.36 | 0.510 | |  | 47.83 | | 12.48 | 48.83 | | 11.75 | 0.889 |
| DSM Scales |  |  |  |  |  |  |  |  |  |  |  | |  |  | |  |  | |  |  |
| Depressive Problems | 52.67 | 4.62 | 50.80 | 1.79 | 0.562 |  | 54.04 | 4.77 | 54.64 | 5.22 | 0.684 | |  | 53.83 | | 7.60 | 54.17 | | 6.59 | 0.937 |
| Anxiety Problems | 52.67 | 4.62 | 52.80 | 6.26 | 0.974 |  | 54.08 | 5.48 | 54.50 | 4.87 | 0.784 | |  | 55.33 | | 4.63 | 53.17 | | 5.00 | 0.454 |
| Autism Spectrum Problems | 53.67 | 6.35 | 51.40 | 2.61 | 0.606 |  | 55.00 | 7.64 | 53.36 | 4.49 | 0.370 | |  | 53.33 | | 3.67 | 53.17 | | 5.85 | 0.954 |
| Attention-Deficit/Hyperactivity Problems | 55.33 | 8.39 | 51.40 | 2.61 | 0.505 |  | 56.72 | 10.75 | 56.18 | 7.41 | 0.845 | |  | 55.17 | | 6.04 | 55.17 | | 7.14 | 1.000 |
| Oppositional Defiant Problems | 55.00 | 6.25 | 50.60 | 1.34 | 0.346 |  | 55.40 | 7.06 | 53.41 | 4.07 | 0.237 | |  | 55.33 | | 6.65 | 53.50 | | 6.32 | 0.635 |
| TRF, Teacher’s Report Form; SF, serum ferritin; Hb, haemoglobin | | | | | | | | | | | | | | | | | | | | |
| Statistically significant associations are highlighted in bold. | | | | | | | | | | | | | | | | | | | | |
